# Visual Sexual Stimuli—Cue or Reward? A Perspective for Interpreting Brain Imaging Findings on Human Sexual Behaviors

**DOI:** 10.3389/fnhum.2016.00402

**Published:** 2016-08-15

**Authors:** Mateusz Gola, Małgorzata Wordecha, Artur Marchewka, Guillaume Sescousse

**Affiliations:** ^1^Swartz Center for Computational Neuroscience, Institute for Neural Computations, University of California San DiegoSan Diego, CA, USA; ^2^Institute of Psychology, Polish Academy of SciencesWarsaw, Poland; ^3^Laboratory of Brain Imaging, Neurobiology Center, Nencki Institute of Experimental Biology of Polish Academy of SciencesWarsaw, Poland; ^4^Donders Institute for Brain, Cognition and Behavior, Radboud UniversityNijmegen, Netherlands

**Keywords:** visual sexual stimuli, neuroimaging, compulsive sexual behaviors, behavioral addictions, incentive salience, reinforcement learning, sexual behavior

## Abstract

There is an increasing number of neuroimaging studies using visual sexual stimuli (VSS), especially within the emerging field of research on compulsive sexual behaviors (CSB). A central question in this field is whether behaviors such as excessive pornography consumption share common brain mechanisms with widely studied substance and behavioral addictions. Depending on how VSS are conceptualized, different predictions can be formulated within the frameworks of Reinforcement Learning or Incentive Salience Theory, where a crucial distinction is made between *conditioned* and *unconditioned* stimuli (related to reward anticipation vs. reward consumption, respectively). Surveying 40 recent human neuroimaging studies we show existing ambiguity about the conceptualization of VSS. Therefore, we feel that it is important to address the question of whether VSS should be considered as conditioned stimuli (cue) or unconditioned stimuli (reward). Here we present our own perspective, which is that in most laboratory settings VSS play a role of *reward*, as evidenced by: (1) experience of pleasure while watching VSS, possibly accompanied by genital reaction; (2) reward-related brain activity correlated with these pleasurable feelings in response to VSS; (3) a willingness to exert effort to view VSS similarly as for other rewarding stimuli such as money; and (4) conditioning for cues predictive of VSS. We hope that this perspective article will initiate a scientific discussion on this important and overlooked topic and increase attention for appropriate interpretations of results of human neuroimaging studies using VSS.

There is an increasing number of neuroimaging studies using visual sexual stimuli (VSS, Figure 1A). VSS are often used as pleasant, arousing stimuli that have an intrinsic positive value (see Wierzba et al., 2015). Brain reactivity triggered by VSS is often interpreted within popular theoretical frameworks describing learning processes or motivated behavior such as Reinforcement Learning (Sutton and Barto, 1998; Botvinick et al., 2009) or Incentive Salience Theory (Robinson and Berridge, 1993; Berridge, 2012). Importantly, these theories make a key distinction between *conditioned stimuli (CS)* and *unconditioned stimuli (UCS)*, which are related to reward anticipation/wanting vs. reward consumption/liking, respectively. Accordingly, it is important to make explicit whether VSS play a role of CS or UCS, i.e., whether they are incentive cues predicting an upcoming reward, or whether they are rewarding by themselves. This issue has been surprisingly overlooked in past studies, despite its important implications. We reviewed 40 human studies published between 2013 and 2016, using VSS in combination with neuroscience methods (fMRI, EEG, ERP, PET, MEG or TMS; Figure 1B):

- Nine studies described VSS as cues/CS: (Minnix et al., 2013; Politis et al., 2013; Steele et al., 2013; Kühn and Gallinat, 2014; Oei et al., 2014; Voon et al., 2014; Wetherill et al., 2014; Prause et al., 2015; Seok and Sohn, 2015).
- Sixteen studies described VSS as rewards/UCS: (Costumero et al., 2013, 2015a,b; Graf et al., 2013; Klucken et al., 2013, 2015, 2016; Sescousse et al., 2013a; Cassidy et al., 2014; Li et al., 2014; Mascaro et al., 2014; Oei et al., 2014; Lee et al., 2015; Banca et al., 2016; Brand et al., 2016; Schöne et al., 2016).
- One study described VSS as both as CS and UCS: (Oei et al., 2014).
- Fifteen studies did not use any such labels: (Abler et al., 2013; Chung et al., 2013; Habermeyer et al., 2013; Hernández-González et al., 2013; Sylva et al., 2013; Wehrum et al., 2013; Borg et al., 2014; Prause et al., 2014; Kim and Jeong, 2013, 2014; Wehrum-Osinsky et al., 2014; Flaisch et al., 2015; Amezcua-Gutiérrez et al., 2016; Kim et al., 2016; Knott et al., 2016).

**Figure 1:**
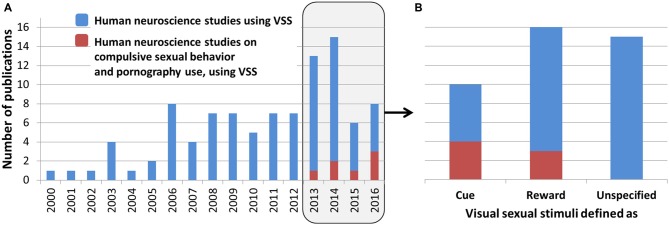
**(A)** Blue bars indicate the number of human studies using neuroscience methods (fMRI, EEG, ERP, PET, MEG or TMS) and visual sexual stimuli (VSS) published between 2000 and 2016 according to PubMed (accessed on March 31st 2016). Red bars indicate the number of neuroscience studies on compulsive sexual behaviors (CSB): 1 in 2013 (Steele et al., 2013), 2 in 2014 (Kühn and Gallinat, 2014; Voon et al., 2014), 1 in 2015 (Prause et al., 2015), and 3 in 2016 (Banca et al., 2016; Brand et al., 2016; Klucken et al., 2016). **(B)** Number of studies published between 2013 and 2016 interpreting VSS as cue, reward or none of these labels (unspecified). Note that in Oei et al. (2014) VSS were defined both as “reward cues” and “rewarding stimuli”, so it was counted in both categories “Cue” and ”Reward”.

The Incentive Salience Theory framework, proposed by Robinson and Berridge (1993), distinguishes two basic components of motivated behavior—“wanting” and “liking”. The latter is directly linked to the *experienced* value of the reward (UCS), while the former is related to the *expected* value of the reward, often carried by a predictive cue (CS). Studies on substance and gambling addiction show that learned cues (CS) related to addiction evoke increased responses in the ventral striatum as well as increased motivated behavior (i.e., shorter reaction times, higher accuracy) among addicted individuals, while responses to the reward itself remain unchanged or undergo blunting over time (Berridge, 2012; Robinson et al., 2015).

Thus, the conceptualization of VSS as cues or rewards in experimental designs is not just a semantic debate, because it has important consequences for the interpretation of neuroimaging results. One important consequence is on the emerging field of neuroscientific research on compulsive sexual behaviors (CSB; Love et al., 2015; Kraus et al., 2016a,b; Figure 1). A central question in this field is whether CSBs (such as excessive pornography consumption Gola et al., 2016a,b) share common brain mechanisms with widely studied substance and behavioral addictions (Love et al., 2015; Gola and Potenza, 2016; Gola et al., 2016c; Kraus et al., 2016b). Depending on how VSS are conceptualized, different predictions can be formulated. If one assumes that VSS play a role of cue, then increased ventral striatal reactivity among subjects with CSB (in comparison with controls) would speak in favor of the addiction hypothesis, while under the assumption that VSS play a role of reward, it is the opposite result (decreased ventral striatal reactivity) that would speak in favor of the same hypothesis. Therefore we feel that it is important to address the question of whether VSS should be considered as cues (CS) or rewards (UCS) in human studies. Here we present our own perspective, hoping that it will initiate a scientific discussion on this topic.

To answer this question we think it is important to distinguish the meaning of VSS in real life vs. in the laboratory setting (Figure 2). In many real life situations, VSS such as the naked body of a sexually attractive partner increase sexual arousal and lead to approach behaviors initializing dyadic sexual activity and ending with orgasm (Georgiadis and Kringelbach, 2012; Gola et al., 2015a). In this case, we argue that VSS play a role of cue (CS), while orgasm plays the role of (primary) reward (UCS). The reasoning is similar in most cases of solitary sexual activity. Most common VSS are pornographic videos or photos (cue/CS), which increase sexual arousal, and lead to masturbation ending with orgasm (reward/UCS). In contrast, during laboratory experiments, subjects are usually not allowed to initiate any sexual activity (such as masturbation) and natural UCS—orgasm—is unavailable. Even if subjects would be allowed to masturbate during the study, laboratory conditions are far less comfortable than the usual context of pornography consumption or dyadic sexual activity. Thus, individuals participating in laboratory experiments do not expect any other reward than being exposed to VSS. Therefore, we posit that in laboratory setting VSS play a role of reward (UCS; Figure 2). The conceptualization of VSS as rewards in the context of laboratory experiments comes with several predictions. Among healthy subjects we should observe: (1) experience of pleasure while watching VSS, possibly accompanied by genital reaction; (2) reward-related brain activity correlated with these pleasurable feelings in response to VSS; (3) a willingness to exert effort to view VSS similarly as for other rewarding stimuli such as money; and (4) conditioning of cues (CS) predictive of VSS. Below we review evidence supporting these predictions.

**Figure 2:**
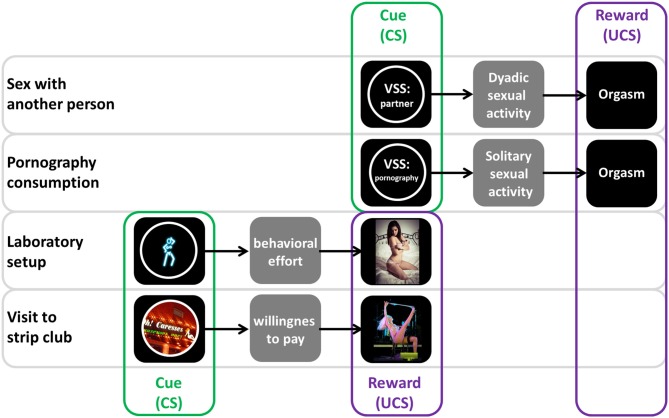
**Visual sexual stimuli (VSS) act as cues in real life, but rewards in the lab.** According to our perspective, in most real life situations (such as sexual activity with partner or solitary pornography consumption) VSS such as the naked body of a sexually attractive partner or pornographic content play a role of cue (CS). VSS increase sexual arousal and lead to behaviors initializing dyadic or solitary sexual activity and ending with reward—i.e., orgasm (UCS). In contrast, in most laboratory settings sexual activity and orgasm are unavailable. We claim that VSS then play a role of reward (UCS), similarly to some real life situations such as the visit to a strip club. In such contexts, individuals do not expect any other reward than being exposed to VSS, and are willing to exert effort or pay money to receive desired VSS, while being susceptible to conditioning for cues predictive of these VSS. For the purpose of illustration of our ideas this figure presents a simplified representation of real life where other scenarios of VSS use are possible, i.e., pornography consumption may lead to dyadic sexual activity or vice versa. Credits of sample photos: Lies Thru a Lens; Strip club in Montreal, Quebec, in Saint Henri borough; Lola Bel Aire, striptease from Miss Exotic World 2008, CC BY 2.0. For license terms see: CC BY 2.0 (https://creativecommons.org/licenses/by/2.0/).

In studies collecting hedonic ratings of VSS, subjects consistently report that watching VSS is a subjectively pleasurable experience when these match the subjects’ sexual preferences (Chivers and Bailey, 2005; Rupp and Wallen, 2009; Jacob et al., 2011; Wierzba et al., 2015). In addition, these hedonic ratings have been shown to be accompanied by genital reactions as measured by penile plethysmography in male participants (Stoléru et al., 1999; Redouté et al., 2000; Ferretti et al., 2005). Erectile reaction among males takes some time so it is easier to observe it with long-lasting VSS such as videos or long presentations of pictures (Ferretti et al., 2005), however even brief presentations of static sexual photographies are related with subjective pleasure and arousal (Ferretti et al., 2005; Wierzba et al., 2015).

Many studies have shown that passive VSS viewing evokes ventral striatum activity (Arnow et al., 2002; Stark et al., 2005; Sabatinelli et al., 2007; Demos et al., 2012; Georgiadis and Kringelbach, 2012; Stoléru et al., 2012; Wehrum-Osinsky et al., 2014). It is difficult to assess whether striatal activity reflects cue related *wanting* or reward related *liking* in these studies given that the ventral striatum is known to respond to both appetitive cues (CS) and rewards (UCS; Flagel et al., 2011; Liu et al., 2011) However, the observed correlation between striatal activity and hedonic ratings triggered by VSS in various studies (Walter et al., 2008; Sescousse et al., 2010, 2013b) favors the hypothesis that VSS act like rewarding stimuli. In this respect, VSS play a similar role as monetary rewards: they activate similar brain areas including the ventral striatum, and trigger comparable hedonic reactions and motivated behaviors (Sescousse et al., 2010, 2013b, 2015). The main difference is that VSS are primary rewards (i.e., they have an intrinsic and innate reward value), whereas money is a secondary reward (whose value is learned by exchange against other rewards). This difference leads to a partially different mapping onto the brain reward system, and different strengths of activation (Sescousse et al., 2010, 2013b, 2015).

Even though most studies using VSS have used passive viewing paradigms, a few investigations have employed more advanced experimental designs aiming to measure participants’ willingness to exert effort towards VSS. In a series of studies, we have used a modified version of the monetary incentive delay task (Knutson et al., 2001) to include VSS (Sescousse et al., 2010, 2013a, 2015; Gola et al., 2015b, 2016c). In this task subjects see two types of cues that are predictive of either VSS or monetary gains. These cues are followed by a discrimination task in which subjects have to press the correct button (out of two) within a time limit of 1 s. The receipt of a monetary gain or VSS is partly dependent on their performance on this task, such that reaction times can be interpreted as an indirect measure of the motivation to obtain these rewards. Importantly, cues predicting VSS elicit similar reaction times as those predicting monetary rewards, demonstrating that participants are willing to exert effort to view VSS, and that their motivation is similar for both rewards (Sescousse et al., 2010). This willingness to exert effort, which is a hallmark of reward (Thorndike, 1965), has been observed in other studies using effort (but also delay) discounting paradigms with VSS (Prévost et al., 2010). In addition, we have shown that individual differences in the effort exerted for money vs. VSS is strongly correlated with the relative brain activity evoked by corresponding cues in the ventral striatum (Sescousse et al., 2015; Gola et al., 2016c). This precise fine-tuning of brain activity and reaction times by VSS predicting cues further confirms that VSS have intrinsically rewarding properties.

Finally, recent studies have shown that abstract CS (such as colorful patterns or dots) associated with VSS maintain their incentive salience even when they are not predictive of VSS anymore (Banca et al., 2016; Klucken et al., 2016). In the study by Banca et al. (2016), abstract visual patterns acquired positive predictive value (CS+) or neutral predictive value (CS−) by being repetitively paired with VSS or neutral stimuli, respectively. In the following phase of the experiment, subjects had to make choices between those CS and novel abstract stimuli, while both CS were now paired with increased chances of monetary gains (but not VSS anymore). Despite both CS having equal chances of leading to monetary gains, CS+ were chosen more frequently than CS− on average (mostly by subjects with CSB), demonstrating the strong rewarding properties of VSS.

As we have shown above, there is a consistent body of evidence supporting our view that in laboratory settings VSS play a role of reward rather than cue. Moreover, even in everyday life VSS do not always play a role of cue for sexual activity and orgasm. Long before the development of photography people have liked art such as sculptures and paintings depicting nudity. Perhaps (similarly to modern times) this type of art was a source of pleasure rather than cue for sexual activity. In the era of photography people showed willingness to pay for pictures and videos with erotic and pornographic content, then internet technology provided everyone with easy and free access to a whole variety of VSS (Cooper, 1998). Perhaps most of contemporary VSS (such as internet pornography) play a role of cue for solitary or dyadic sexual activity, but in some cases VSS are sought after for themselves, again demonstrating their intrinsic rewarding value. A good example in everyday life are calendars with erotic pictures, that people buy and expose in their workplace or at home. Similarly, the popularity of strip clubs, in which people are willing to pay to watch nude dancers with whom they are not allowed to engage in sexual activity, illustrate the potency of VSS as hedonic stimuli (Figure 2).

Based on the above arguments, we argue that VSS play a role of reward—rather than cue—in most experimental setups in which sexual activity and climax experience are unavailable. As we outlined above, viewing VSS is a pleasurable experience that people are willing to work and wait for (Prévost et al., 2010), and activates the same brain reward regions as monetary gains (Sescousse et al., 2010, 2013a, 2015; Gola et al., 2015b, 2016c). Moreover neutral stimuli associated with VSS through Pavlovian conditioning acquire incentive value (Sescousse et al., 2010, 2013a, 2015; Banca et al., 2016; Gola et al., 2016c; Klucken et al., 2016). This conceptualization of VSS as rewards rather than cues calls for the re-examination—and possibly re-interpretation—of the results reported in earlier studies defining VSS as cues. Certainly it may have a strong impact on the interpretation of neuroimaging studies investigating neurobiological similarities between CSB and addiction; for instance, based on the popular Incentive Salience Theory framework, one would expect opposite ventral striatal reactivity for VSS depending on whether they are conceptualized as a cue or reward (as an example of such ambiguous interpretation see: Prause et al., 2015, 2016; see also Gola, 2016 for discussion). If in most of experimental setups, as we argue, VSS play a role of reward, then diminished (rather than increased) ventral striatal reactivity to VSS in individuals with problematic pornography use (Gola et al., 2016a) would speak in favor of the addiction hypothesis (Robinson et al., 2015). We would expect this to be accompanied by increased ventral striatum activations for CS that are predictive of VSS, as well as increased effort or shorter reaction times to gain access to these VSS. In future studies, we hope that the role played by VSS in the specific protocols that are used will receive increased attention, and that appropriate interpretations of results will be made accordingly.

## Additional Information

### Method of Study Selection

We searched the Pubmed database from 2000 to 2016 to identify neuroscience publications (key words: fMRI, EEG, ERP, PET, MEG or TMS) with VSS (keywords: VSS, sexual stimuli, erotic stimuli, sexual pictures, erotic pictures, sexual images, erotic images, sexual videos, erotic videos). Only full peer-reviewed publications were selected (no conference abstracts). For studies published between 2013 (year of first publication on problematic pornography use) and 2016 we categorized them into three categories depending on whether VSS were described as: (1) “cue/CS”; (2) “reward/rewarding stimuli/UCS”; and (3) otherwise.

### Related Issues

Here we want to highlight several issues which, if properly investigated, may provide valuable information in the debate on the interpretation of studies using VSS and help to extend the significance of future research.

One of the crucial points is to examine the difference in behavioral and neural responding when VSS are used as cues vs. rewards. It could be done by comparing two experimental conditions in which VSS play a role of reward (most of current experimental settings) or cue (settings allowing subjects to climax during or after the study).

Another interesting hypothesis is that behavior and brain activaty elicited by VSS in typical experimental settings may partly reflect inhibitory control. This inhibitory control may be removed at the end of the experiment, after which subjects may start seeking sexual encounters or initiate solitary sexual activity. For instance, an old behavioral study by Brown et al. (1976) has shown that among heterosexual males, VSS viewing in the laboratory induced masturbation in 24.5% of the subjects on the day of the experiment, while on other days only 12.5% of them engaged in masturbation. This observation suggests that for a fraction of the subjects, watching VSS in the laboratory may have been a cue eliciting sexual motivation that had to be inhibited. To examine such a possibility it would be important to control for sexual activity following experimental studies. Furthermore it raises several questions: does this subgroup differ from other participants, i.e., in terms of sexual arousability (Gola et al., 2015a)? And if so, than does it affect brain activity?

We hope that these questions will inspire investigators and will be addressed in future studies.

## Author Contributions

All authors discussed the idea. MG prepared figures. MW and MG did a review of literature. MG and GS wrote the manuscript. AM and MW commented on the manuscript.

## Funding

MG was supported by Opus grant from National Science Centre in Poland (2014/15/B/HS6/03792; MG) and scholarship of Ministry of Science and Higher Education of Republic of Poland (469/STYP/10/2015); MW was supported by Opus grant from National Science Centre in Poland (2014/15/B/HS6/03792; MG); GS was supported by a Veni grant from the Netherlands Research Organization (NWO, ref no. 016.155.218).

## Conflict of Interest Statement

The authors declare that the research was conducted in the absence of any commercial or financial relationships that could be construed as a potential conflict of interest. The reviewers RS and TK declared their shared affiliation, and the handling Editor states that the process nevertheless met the standards of a fair and objective review.
